# From himachalenes to trans-himachalol: unveiling bioactivity through hemisynthesis and molecular docking analysis

**DOI:** 10.1038/s41598-023-44652-z

**Published:** 2023-10-17

**Authors:** A. Faris, Y. Edder, I. Louchachha, I. Ait Lahcen, K. Azzaoui, B. Hammouti, M. Merzouki, A. Challioui, B. Boualy, A. Karim, G. Hanbali, S. Jodeh

**Affiliations:** 1 Equipe de Chimie de Coordination et Catalyse, Département de Chimie, Faculté des Sciences Semlalia, Université Cadi Ayyad, B.P. 2390, 40001 Marrakech, Morocco; 2 Laboratory of Engineering, Electrochemistry, Modeling and Environment, Faculty of Sciences, Sidi Mohamed Ben Abdellah University, 30000 Fez, Morocco; 3 Euro-Mediterranean University of Fes, B.P. 15, 30070 Fez, Morocco; 4 Laboratoire de Chimie Appliquée et Environnement - Equipe Chimie Organique Macromoléculaire et Phytochimie, Faculté des Sciences, Université Mohammed Ier, 60000 Oujda, Morocco; 5 Environmental Sciences and Applied Materials Research Team, Multidisciplinary Research and Innovation Laboratory, Polydisciplinary Faculty of Khouribga, Sultan Moulay Slimane University of Beni Mellal, B.P. 145, 25000 Khouribga, Morocco; 6 Department of Chemistry, An-Najah National University, P.O. Box 7, Nablus, Palestine

**Keywords:** Biochemistry, Drug discovery, Plant sciences

## Abstract

In this study, we report the first total hemisynthesis of trans-himachalol sesquiterpene, a stereoisomer of the natural cis-himachalol isolated from Cedrus atlantica essential oils, from himachalenes mixture in five steps. Reactions conditions were optimized and structures of the obtained compounds were confirmed by IR, mass spectra, ^1^H, and ^13^C NMR. The synthesized compounds were investigated for potential activities on various isolated smooth muscles and against different neurotransmitters using molecular docking. The results show that the synthesized compounds display high affinities towards the active site of the protein 7B2W and the compounds exhibit promising activities on various isolated smooth muscles and against different neurotransmitters.

## Introduction

The essential oil of Atlas cedar (Cedrus atlantica) is mainly composed of three sesquiterpenic bicyclic hydrocarbons: β, γ and α-himachalene **(1)**, **(2)** and **(3)** respectively, together, they can make up to 70% of the oil composition. The other 30% is composed of oxygenated terpenes such as atlantones, himachalene oxide and cis-himachalol^1,2^.

This essential oil has been subjected to different pharmacological and phytochemical studies revealing, among others, its anti-inflammatory, analgesic, antibacterial, anti-fungal, anti-spasmodic and anti-cancer effects^3–5^.

**Figure 1:**
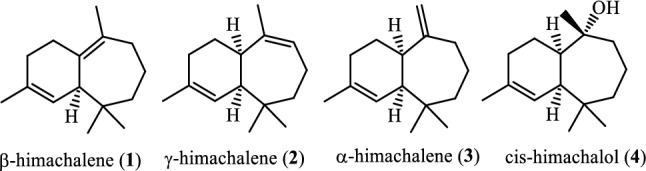
Main components of cedarwood essential oil**.**

Cis-himachalol (4) (Fig. 1) has been identified as a minor constituent of the essential oil of Atlas cedar as reported in previous studies^6^. It was originally isolated and characterized in 1968 by S.C. Bisarya and Sukh Dev from Cedrus deodara^7^. This compound is responsible for many of the essential oil pharmacological activities. It has demonstrated a significant activity as an anti-spasmodic; several pharmacological studies involving cis-himachalol on various isolated smooth muscles, including Guinea pig ileum, Rabbit jejunum and Rat uterus as well as an antagonist for different neurotransmitters such as acetylcholine, histamine, and serotonin have indicated its antispasmodic activity, resembling that of papaverine^8^.

Other studies have revealed the effectiveness of himachalol as an insecticidal against the seed beetle and the housefly^9^. Furthermore, at low concentrations, it has demonstrated in vitro inhibitory activity against Aspergillus fumigatus, a fungus belonging to the Aspergillus genus, known for causing severe infections in humans and birds^10^. Additionally, Recent published research highlighted that cis-himachalol possesses potent cytotoxic activity against several human cancers, including brain, colon, and ovarian cancer^11,12^. Surprisingly, albeit the pharmacological significant activities of cis-himachalol, to the best of our knowledge, a total synthesis of this natural sesquiterpene has never been reported.

Herein, as a part of our ongoing study targeting himachalenes as a starting material for the development of new functionalized sesquiterpenes of highly added value^13–19^, we report a first total synthesis of trans-himachalol (**5)** from himachalenes, readily and abundantly available sesquiterpenes, in five steps.

## Experimental

### Material

All reagents and solvents used were purchased from commercial sources and used as received without further purification (Aldrich, Acros). NMR studies were performed on a Bruker Avance 300 MHz spectrometer in CDCl_3_ solution, chemical shifts are given in ppm relative to external TMS and coupling constant (J) in Hz. Liquid chromatography was performed on silica gel (Merk 60, 220–440 mesh; eluent: hexane/ethyl acetate). The reaction mixtures were analyzed on a Trace GC Thermo Finnigan chromatograph equipped with FID, using capillary columns BP (25 m, 0.25 mm, SGE).For MS detection, the ionization was performed on an ISQ LT single quadruple mass spectrometer in positive EI modes using a mass scan range of 50–400 Da. Infrared (IR) spectrum VERTEX 70 in the range 4000–400 cm^−1^region.

### Preparation methods of different synthesized products

#### Himachalene dihydrochloride (9)

30 g (0.147 mol) of a mixture of α-, β-, and γ-himachalenes (1, 2 and 3) (obtained by a fractional distillation under reduced pressure. Temperature: 100 °C, Pressure: 0.1 mmHg) were dissolved in 60 mL of anhydrous acetic acid. Gaseous HCl was bubbled at 0 °C for 20 h. The resulting colored product was allowed to crystallize in a freezer at − 5 °C overnight. After vacuum filtration, the residue was purified by recrystallization in a toluene-hexane solvent mixture (1:5). The obtained crystals appear as fine white needles with a mass of 23.6 g (0.085 mol), corresponding to a yield of 58%.

(C_15_H_26_Cl_2_). mp: 118–120 °C; ^1^H NMR (300 MHz, CDCl_3_) δ (ppm): 0.78 (3H, s); 0.90 (3H, s); 1.54 (3H, s); 1.56 (3H, s). ^13^C NMR (75 MHz, CDCl_3_) δ (ppm): 19.58 (CH_3_); 31.96 (CH_3_); 20.42 (CH_2_); 24.57 (CH_2_); 33.35 (CH_3_); 34.62 (CH_3_); 35.27 (C); 39.73 (2CH_2_); 42.87 (CH_2_); 43.09 (CH_2_); 45.12 (CH); 50.11 (CH); 71.08 (C); 75.93 (C).

#### Himachalene monohydrochloride (8)

1g (4.16 mmol) of himachalene dihydrochloride (9) was dissolved in 15 mL of methanol. After complete dissolution, the solution was maintained at 0 °C for 1 h. Then it was vacuum filtrated, which yielded the himachalene monohydrochloride as white crystals, appearing as fine needles, with a mass of 0.6g (2.49 mmol), corresponding to a yield of 60%.

(C_15_H_25_Cl). mp: 49 °C; ^1^H NMR (300 MHz, CDCl_3_) δ (ppm): 0.79 (3H, s); 0.88 (3H, s); 1.63 (3H, s), 4.69 et 4.71 (2H (of = CH_2_, s, s). ^13^C NMR (75 MHz, CDCl_3_) δ (ppm): 22.23 (CH_3_); 24.36 (CH_2_); 30.04 (CH_3_); 31.77 (CH_2_); 33.06 (CH_2_); 34.61 (CH_3_); 35.79 (C); 41.49 (CH_2_); 42.94 (CH_2_); 43.04 (CH_2_); 43.28 (CH); 47.74 (CH); 73.02 (C); 110.29 (= CH_2_); 156.31 (C).

#### Himachalone monohydrochloride (7)

In a solution of 1 g (4.17 mmol) of himachalene monohydrochloride (**8**) in a solvent mixture: acetonitrile; dichloromethane; water (2: 2: 1), 0.02 g (0.069 mmol) of RuCl_3,_ 6H_2_O, and 1.79 g (8.36 mmol) of NaIO_4_ were added, the reaction mixture was stirred at room temperature for 2 days. Periodic samples of 1 mL were extracted with ether (2 × 1 mL), the extract was then washed with distilled water, dried over MgSO_4_ and analyzed by chromatography (GC). This makes it possible to follow the evolution of the reaction over time. At the end of the reaction, the mixture is extracted with ethyl acetate. The organic phase is washed with distilled water (2 × 30 mL), then dried over MgSO_4_, the solvent is then evaporated under reduced pressure. The product (**7)** was purified on silica gel chromatography, eluted with hexane and hexane/EtOAc (90:10), a dark red viscous oil was obtained, mass 1.02 g (4.22 mmol) (yield: 78%).

(C_14_H_23_OCl). IR (neat) max/cm^−1^ 1704,26 (CO carbonyl); MS(m/z) 242.2; NMR^1^H (300 MHz, CDCl_3_) δ (ppm): 0.66 (3H, s); 0.88 (3H,s); 1.16 (1H (of CH), td (13.05 Hz, 2.7 Hz)); 1.39–1.5 (4H (of CH_2_),m) and 1H (of CH_2_), m); 1.57 (3H,s); 1.62 (1H (of CH_2_), m); 1.64–1.78 (1H (of CH_2_) and 1H (of CH_2_), m,m); 1.95 (1H (of CH_2_),m); 1.96 (1H (of CH_2_), m); 1.98 (1H (of CH), m); 2.72(1H (of CH_2_ td (11.4 Hz, 5.5 Hz)); 2.22 (1H (of CH_2_), td (10.2 Hz, 6.6 Hz)). NMR ^13^C (75 MHz, CDCl_3_) δ (ppm): 19.89 (CH_2_); 21.75 (CH_3_); 27.32 (CH_2_);29.29 (CH_3_); 34.34 (CH_3_); 35.64 (C); 39.05 (CH_2_); 40.55 (CH_2_); 41.75 (CH); 41.99 (CH_2_); 42.07 (CH_2_); 54.34 (CH); 72.14 (C); 216.75(C).

#### Himachalone (6)

In a 100 ml flask, we introduced 50 ml of EtOH, 4.28 g (62.1 mmol) of EtONa and 1 g (4.14 mmol) of himachalone monohydrochloride **(7)**. The reaction mixture brought to 45 °C for 8 h. At the end of the reaction, the ethanol was evaporated and the mixture extracted with ethyl acetate and water. The organic phase was washed with distilled water (2 × 20 mL), then dried over MgSO_4_, the solvent then was evaporated under reduced pressure. Compund **(6)** was purified on silica gel chromatography, eluted with hexane and hexane/EtOAc (90:10), a dark red viscous oil was obtained, mass 0.78 g (3.8 mmol) (yield: 92%).

(C_14_H_22_O). IR (neat) max/cm^−1^ 1700,86 (CO carbonyl). MS(m/z) 206,2. NMR ^1^H (300 MHz, CDCl_3_) δ (ppm): 0.71 (3H (of CH_3_), s); 0.91(3H (of CH_3_), s); 1.21 (1H (of CH_2_), m); 1.45 (1H (of CH_2_), m); 1.52 (2H (of CH_2_), m); 1.62 (3H (of CH_3_), s); 1.65 (1H (of CH_2_), m); 1.70(1H (of CH_2_), m); 1.74(1H (of CH_2_), m); 1.96–2.01 (1H (of CH), 1H (of CH_2_) and 1H (of CH_2_), m, m, m); 2.15–2.26(1H (of CH_2_) and 1H (of CH), m, m); 5.32 (1H (of CH), d, 2.7 Hz ). NMR^13^C (75 MHz, CDC_l3_) δ (ppm): 18.98 (CH_3_); 22.11 (CH_2_); 23.43 (CH_3_); 30.61 (CH_3_); 31.06 (CH_2_); 32.22 (CH_2_); 36 (C); 40.12 (CH_2_); 43.22 (CH); 45.71 (CH_2_); 53.42 (CH); 118.68 (CH); 134.41 (C); 217.08(C).

#### Trans-himachalol (5)

A suspension of 0.4 g (16.44 mmol) of Mg in 30 ml of ether was stirred at room temperature, followed by the introduction of 0.71 g (5 mmol) of CH_3_I and 0.1 g (0.39 mmol) of iodine (I_2_). After 30 min, a solution of 1 g (4.85 mmol) of himachalone **(6)** diluted in 20 mL of ether was added dropwise during 30 min. After stirring for 4 h at room temperature, an NH_4_Cl (2 g)/H_2_O solution (20 ml) is added dropwise over 1 h. At the end of the reaction, the mixture was extracted with ethyl ether (2 × 20 mL) and distilled water, the organic layer was dried over MgSO_4_ and the solvent was evaporated under reduced pressure. The product is purified on silica gel chromatography, eluted with hexane/EtOAc (90:10) and hexane/EtOAc (70:30), a dark red viscous oil was obtained, mass 1.032 g (4.65 mmol) (yield: 96%).

(C_15_H_26_O). IR (neat) max/cm^-1^ 3466,27 (OH alcohol); 1696 cm^-1^ (C-O). MS (m/z) 222,1. NMR^1^H (300 MHz, CDCl_3_) δ (ppm): 0.71 (3H of (CH_3 (14)_), s); 0.91 (3H of (CH_3 (15)_), s); 1.17 (3H of (CH_3 (12)_), s); 1.2–1,7 (9H, (4CH_2_ and 1CH)_,_ m); 1.71 (3H of (CH_3 (13)_), s); 1.75–2 (4H of (CH_2_, 1CH and OH), m); 5.53 (1H of (CH_(8)_), d, 10,4 Hz). NMR^13^C (75 MHz, CDC_l3_) δ (ppm): 19,18 (C_(4)_); 22,72 (C_(15)_); 23,51 (C_(14)_); 25,17 (C_(11)_); 29,29 (C_(7)_) 30,13 (C_(1)_); 30,42 (C_(10)_); 35,53 (C_(6)_); 40.01 (C_(12)_); 41,64 (C_(13)_); 44,1 (C_(5)_); 45,84 (C_(3)_); 74,53 (C_(2)_); 121.16 (C_(9)_); 139.62 (C_(8)_).
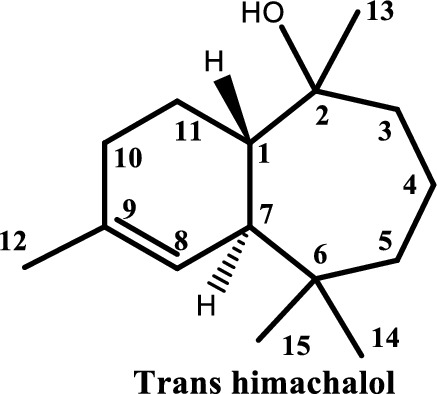


### ADME studies

The ADME (Absorption, Distribution, Metabolism, and Excretion) features of the Schrödinger software's QikProp module were utilized to compare the main compounds derived from himachalenes (trans-himachalol (5), himachalone (6), and himachalone monohydrochloride (7)) to a positive control (Physostigmine). The drawing tool available in the input area enables users to generate, edit, and import 2D structures. Output files can be saved using PDB files, which can be verified using any visualization software. This investigation unveils notable physicochemical characteristics, including molecular weight/size, hydrophobicity, bioavailability, permeability, and polar solubility. Lipinski's rule of five violations was applied to the most thoroughly examined compounds to demonstrate their potential^20^.

### Molecular docking studies and analysis

**Figure 2:**
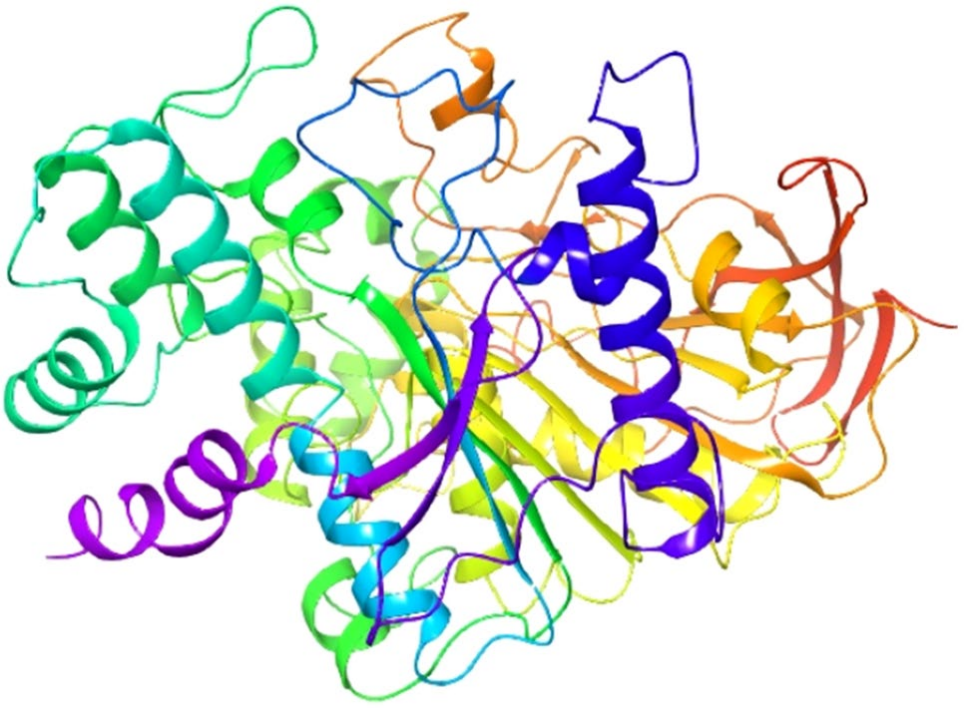
The three-dimensional structure of the protein (PDBID: 7B2W).

The main compounds derived from himachalenes (trans-himachalol (**5**), himachalone (**6**), and himachalone monohydrochloride (**7**)) were synthesized for their activities on various isolated smooth muscles and against different neurotransmitters. The three-dimensional structures (3D) of these compounds, along with the ligand Physostigmine, were obtained from the PubChem database in Structure Data Format (SDF) and used as a positive control for molecular docking studies. The preparation of the compounds and protein structures involved 3D and geometric optimizations, ligand energy minimization, and energy minimization using the OPLS4 force field in Schrödinger 2021–2^21^. Molecular docking was performed on Torpedo californica acetylcholinesterase complexed with UO2 (PDBID: 7B2W) Fig. 2 with a resolution of 2.65 Å^22,23^. The protein structure was prepared using the Protein Preparation Wizard, considering the 3D configuration. Non-required water molecules were removed before the docking assay. Initially, a blind docking approach was used to explore potential binding sites, followed by sequential docking of each ligand to its appropriate best binding site (BBS)^24^. This approach allows the investigation of synergistic or antagonistic interactions between the ligands. Finally, the selected conformation was depicted in a two-dimensional diagram to illustrate the ligand's interaction with active site residues using BIOVIA Discovery Studio, 2021.

## Results and discussion

### IR, mass spectra, ^1^H, and ^13^C NMR Results

**Scheme 1:**
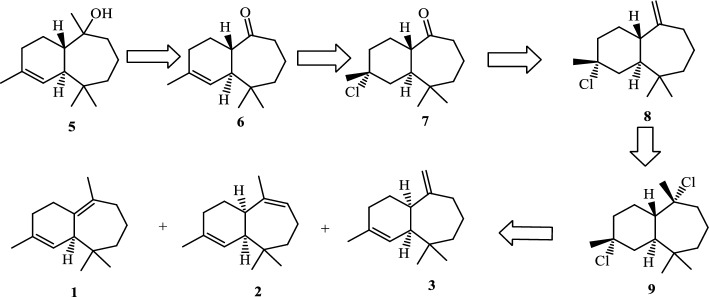
Retrosynthetic Analysis for the Synthesis of trans-himachalol (5).

Our retrosynthetic analysis for the total synthesis of trans-himachalol (**5)** is shown in Scheme 1. The target compound can be synthesized from ketone (**6)** by Grignard reaction. Precursor (**6)** that can be obtained by dehydrohalogenation of himachalone monohydrochloride (**7)**, which can, in turn, be synthesized from himachalene monohydrochloride (**8)** by oxidative cleavage. Compound (**8)** can be prepared from himachalene dihydrochloride by dehydrochlorination of compound (**9)**. Whereas compound (**9)** can be obtained by hydrochlorination of himachalenes mixture (**1**, **2** and **3**).

**Scheme 2:**
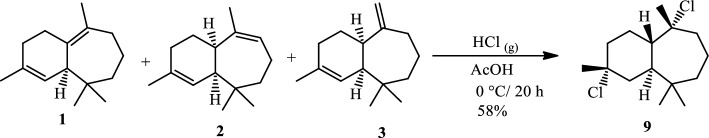
Synthesis of himachalene dihydrochloride.

**Scheme 3:**
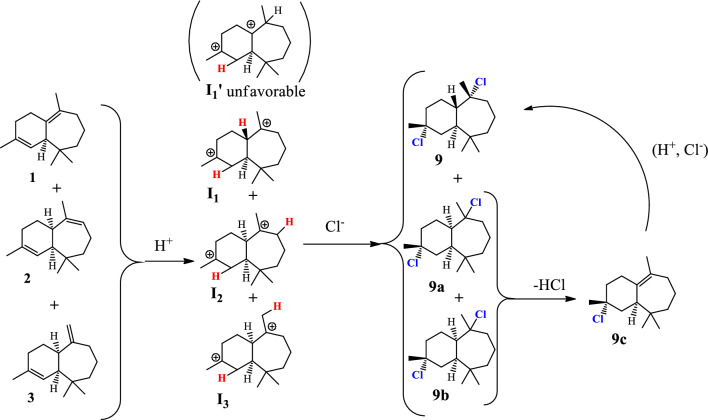
proposed mechanism for the formation of 9 by hydrochloration of 1,2 and 3 mixture.

The synthesis of trans-himachalol **(5)** began with the himachalenes mixture (**1**, **2** and **3**) as the starting material, obtained by a fractional distillation under reduced pressure of the essential oil. The hydrochlorination of himachalenes was conducted as described in the literature (Scheme 2)^25^. Himachalene dihydrochloride (**9**) was obtained in 58% yield. Herein, an inversion of the stereochemistry of one of the carbons joining the two cycles of himachalene skeleton is noticed. In order to explain this inversion, we proposed the following mechanism (Scheme 3).

The addition of H^+^ on the himachalenes **1**, **2** and **3** lead to the formation of the most stable tertiary carbocations **I**_**1**_, **I**_**2**_ and **I**_**3**_ respectively. It is noteworthy, that the addition of H^+^ on the double bond on the seven-membered ring of **1** can lead to the formation of two tertiary carbocations **I**_**1**_ and **I**_**1**_**’**. The carbocation **I**_**1**_**’** is less favorable due to the carbocation planar geometry restrained by the two cycles. The addition of Cl^-^ on carbocations lead to the formation of **9**, **9a** and **9b**, an elimination-addition of HCl on **9a** and **9b** lead to their isomerization and formation of our desired product **9.**

The obtained product (9) is characterized by ^1^H NMR, ^13^C NMR.

**Figure 3:**
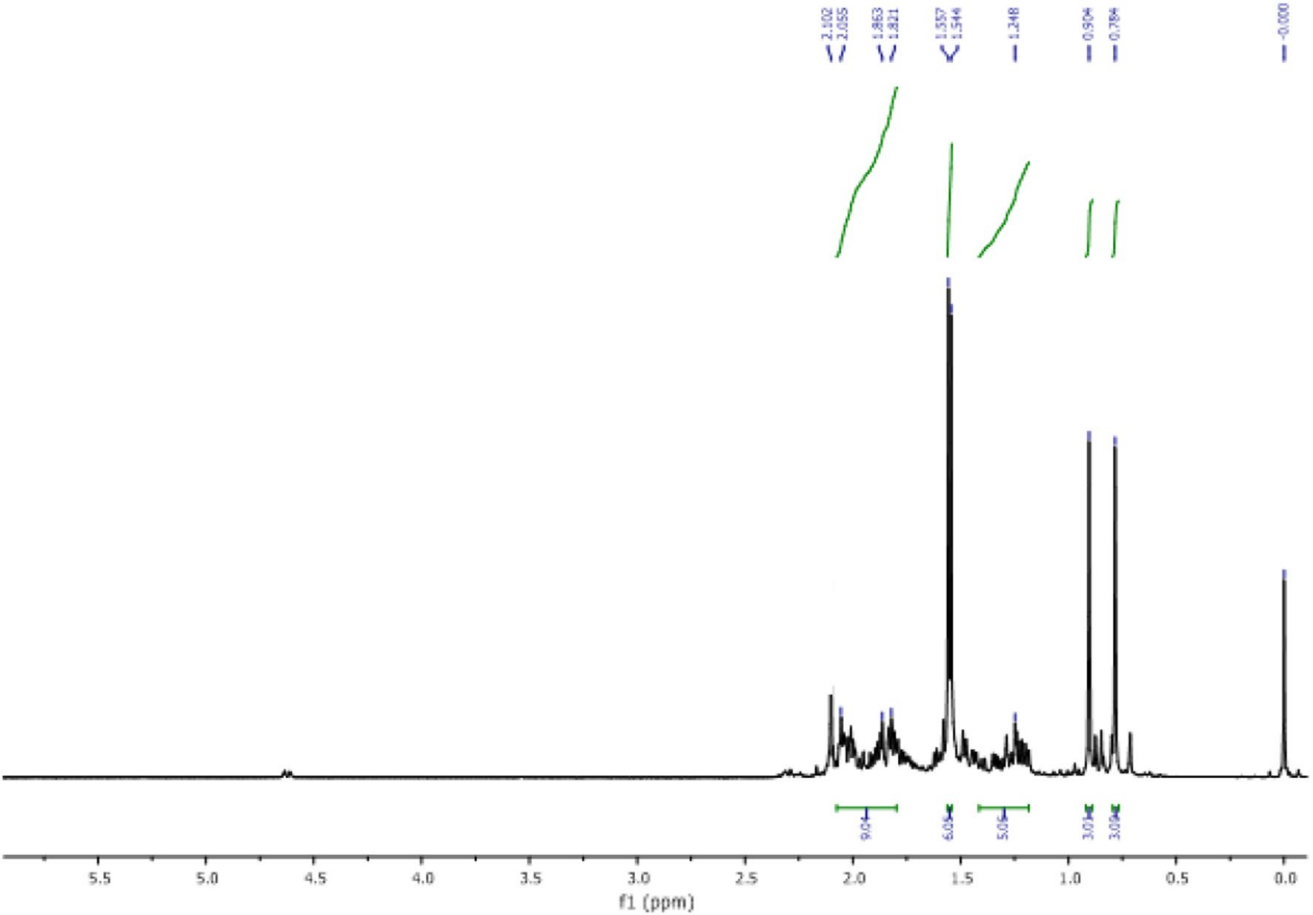
^1^H NMR of himachalene dihydrochloride (9).

In the ^1^H NMR spectrum (Fig. 3), we observe:

- Four signals at 0.78 ppm, 0.90 ppm, 1.54 ppm, and 1.55 ppm corresponding to the characteristic protons of the methyl groups.

**Figure 4:**
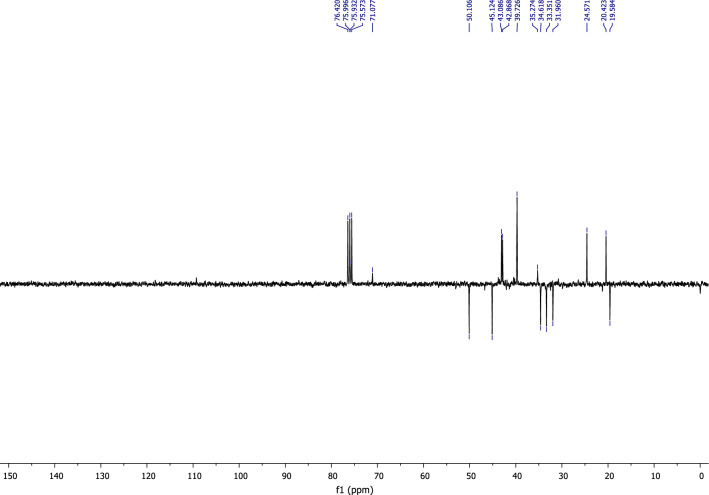
^13^C NMR of himachalene dihydrochloride (9).

According to the analysis of the ^13^C NMR spectrum using APT programming (Fig. 4), it is easy to identify the distinctive peaks of the molecule, notably two peaks at 71,07 ppm and 75,93 ppm corresponding to the quaternary carbons bonded to chlorine atoms.

**Scheme 4:**
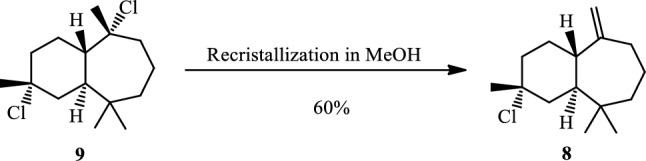
Synthesis of himachalene monohydrochloride.

The recrystallization of himachalene dihydrochloride (**9**) in methanol gives the himachalene monohydrochloride (**8)** in a 60% yield (Scheme 4).

The obtained product (8) is characterized by ^1^H NMR, ^13^C NMR.

**Figure 5:**
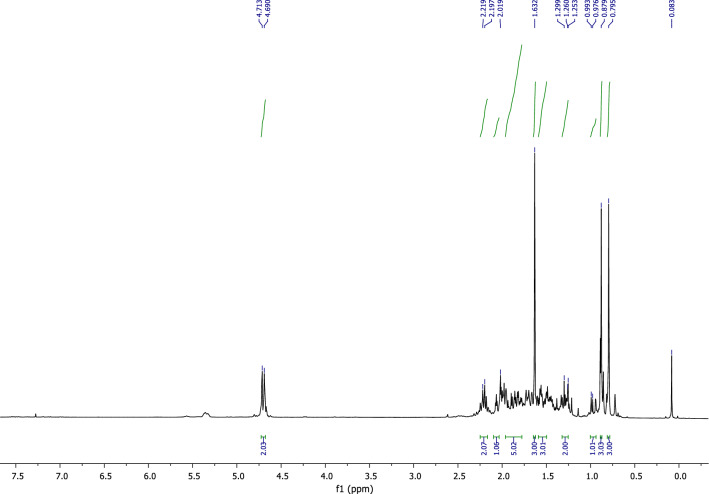
^1^H NMR of himachalene monohydrochloride (8).

In the ^1^H NMR spectrum (Fig. 5), we observe:

- Three signals at 0.79 ppm, 0.87 ppm, and 1.55 ppm correspond to the protons of methyl groups.
- Two singlet peaks at 4.69 ppm and 4.71 ppm correspond to the protons of the methylene group (=CH_2_).

**Figure 6:**
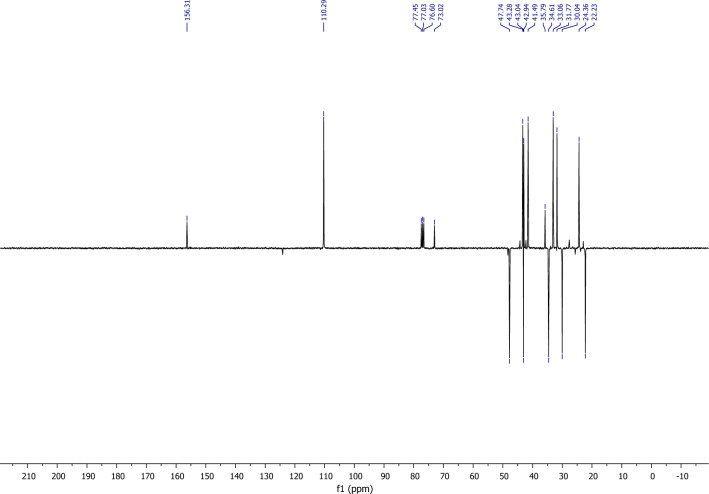
^13^C NMR of himachalene monohydrochloride (8).

According to the analysis of the ^13^C NMR spectrum using APT programming (Fig. 6), it is straightforward to identify the distinctive peaks of the molecule, notably:

- A peak at 71.02 ppm corresponding to the quaternary carbon linked to the chlorine atom.
- Two peaks at 110,29 ppm and 156,31 ppm corresponding, respectively, to the tertiary and quaternary carbons of the molecule's double bond.

**Scheme 5:**
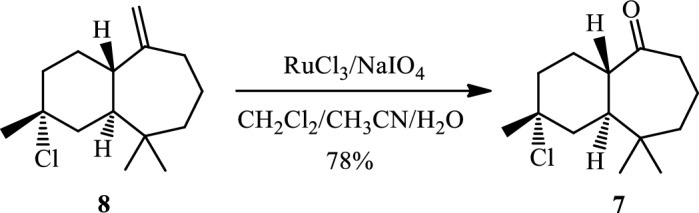
Catalytic oxidative cleavage of himachalene monohydrochloride.

Compound (**(8)** was then converted to the ketone (**7)** by an oxidative cleavage reaction (Scheme 5). This reaction was performed using the RuCl_3_/NaIO_4_ system^26^ in Dichloromethane/Acetonitrile/Water (2/2/1) at room temperature and the desired product **7** was obtained in a good yield (78%).

The obtained product (7) was purified using silica gel column chromatography eluted with hexane/ethyl acetate (90/10) and characterized by ^1^H NMR, ^13^C NMR, and 2D NMR (COSY and HSQC) spectroscopy.

**Figure 7:**
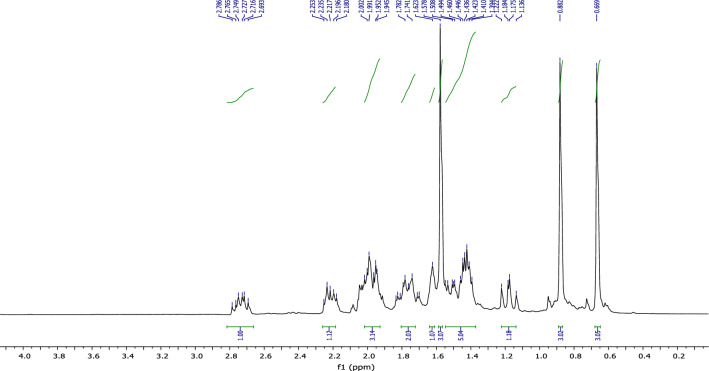
NMR ^1^H (CDCl_3_) of himachalone monohydrochloride (7).

In the ^1^H NMR spectrum (Fig. 7), we observe:

• Three signals at 0.64 ppm, 0.8 ppm, and 1.55 ppm, corresponding to the characteristic methyl groups protons.

• Two signals at 2.18 ppm and 2.72 ppm, which correspond to the two protons of (CH_2_CO).

**Figure 8:**
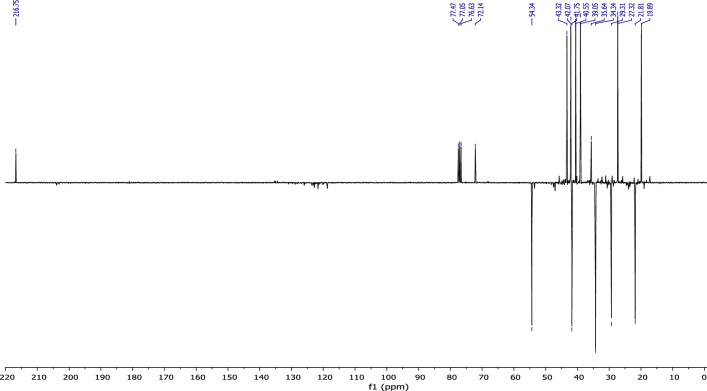
NMR ^13^C (CDCl_3_) of himachalone monohydrochloride (7).

According to the ^13^C NMR spectrum using APT (Fig. 8) pulse sequence, we clearly observe fourteen peaks corresponding to fourteen carbon atoms of the molecule, notably: the appearance of the peak corresponding to the quaternary carbon of the carbonyl group at 216 ppm, and the disappearance of the secondary carbon of the methylene group from the starting material at 110.26 ppm.

**Figure 9:**
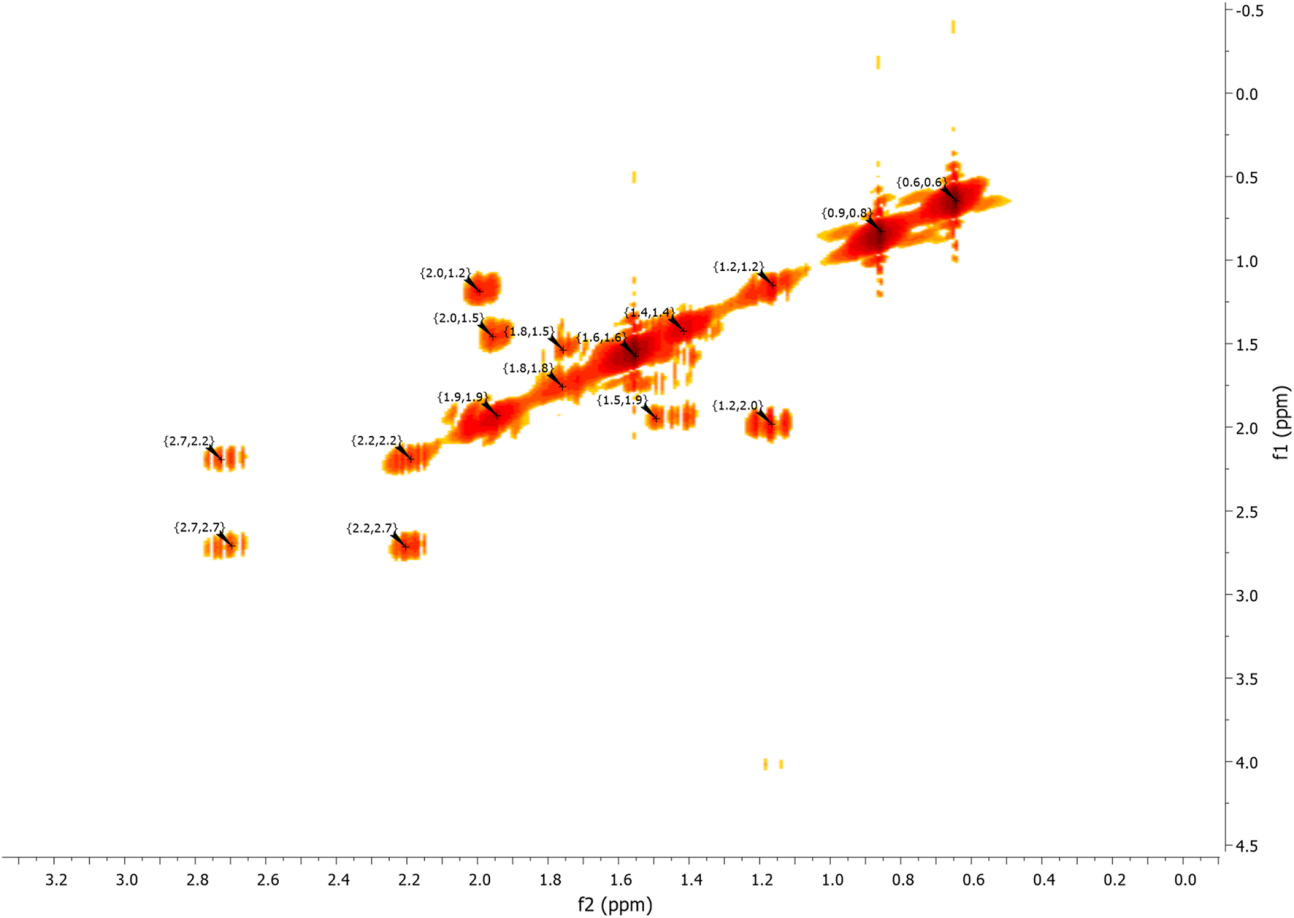
NMR 2D COSY (CDCl_3_) of himachalene monohydrochloride (7).

**Figure 10:**
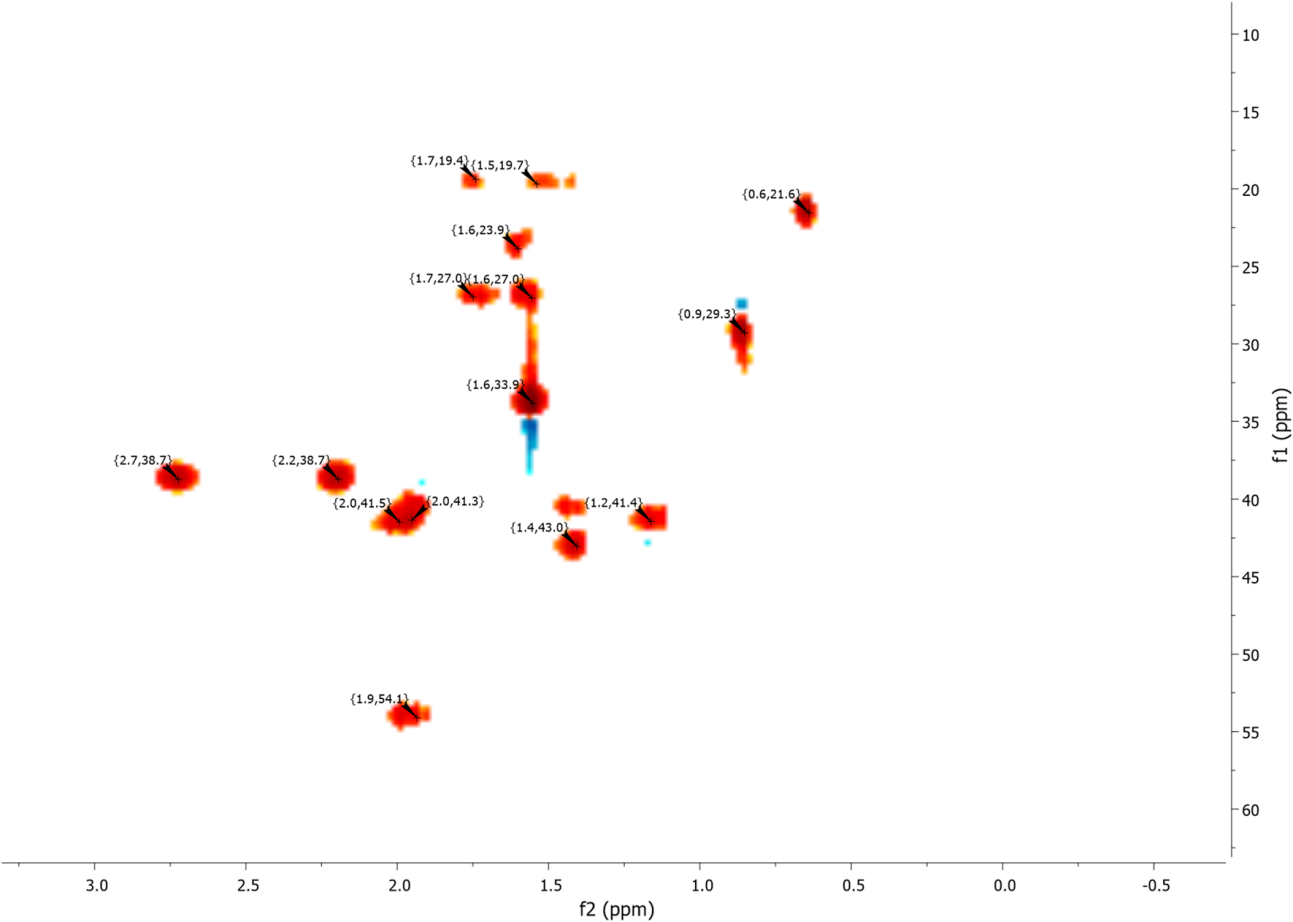
NMR 2D HSQC (CDCl_3_) of himachalene monohydrochloride (7).

^1^H NMR (Fig. 7) and 2D NMR experiments utilizing COSY (Fig. 9) and HSQC (Fig. 10) pulse sequences reveal, among other observations, that the alpha proton of the ketone group, positioned at the intersection of the two cycles, is observed at 1.16 ppm as a triplet of doublets featuring coupling constants of (13.05 Hz, 2.7 Hz). This verifies the trans stereochemistry between the protons at the cycle junction. Specifically, the aforementioned proton engages in interactions with neighboring protons in the trans configuration, resulting in the manifestation of a triplet (J_3_ = 13.05 Hz). Moreover, it is subject to coupling with the adjacent proton in the cis configuration, resulting in a doublet (J_3_ = 2.7 Hz) within the spectrum.

**Figure 11:**
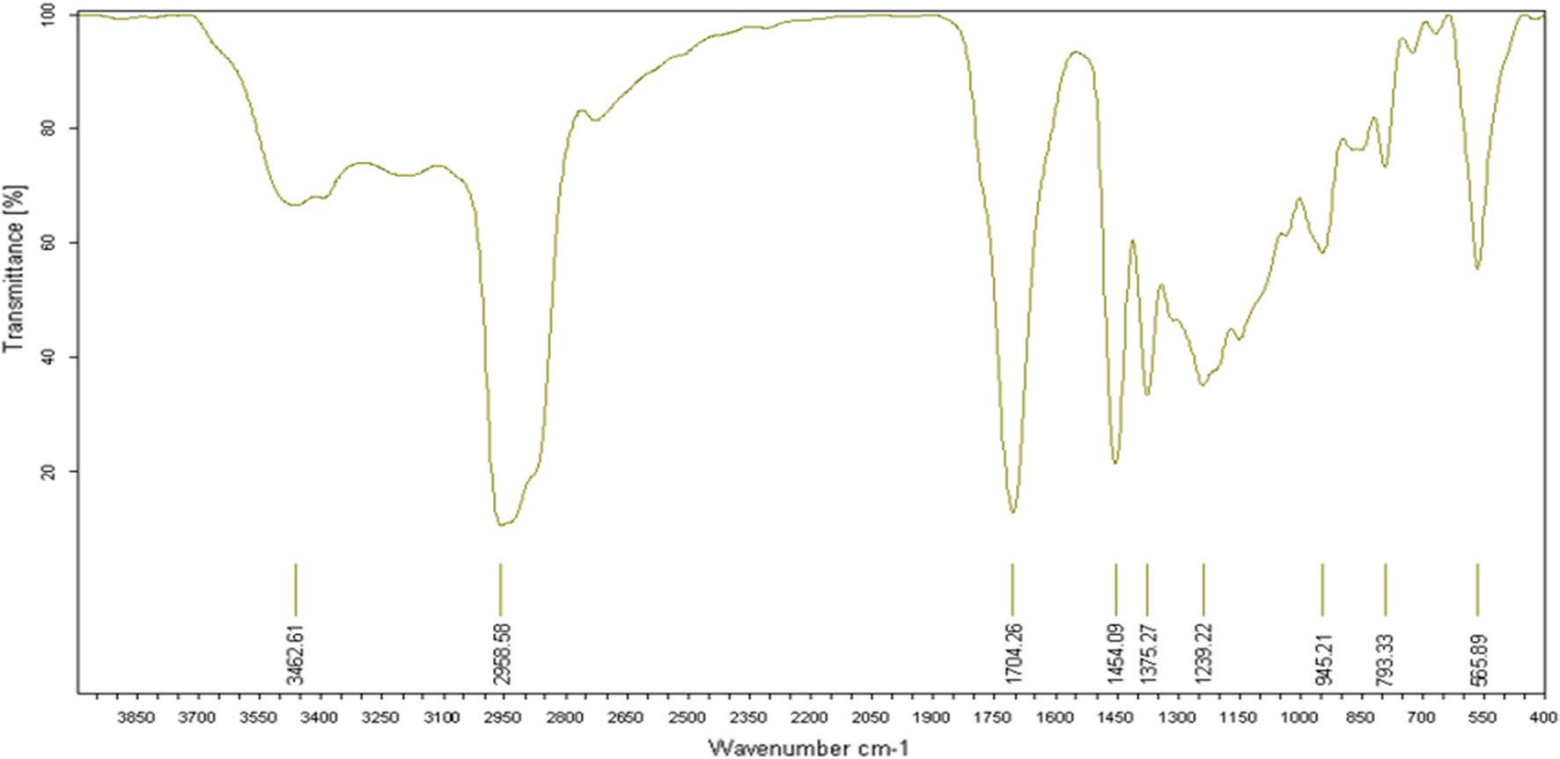
IR spectrum of himachalene monohydrochloride (7).

The IR analysis (Fig. 11) reveals a broad and intense band characteristic of the ketone functional group at 1704.26 cm^-1^.

**Scheme 6:**
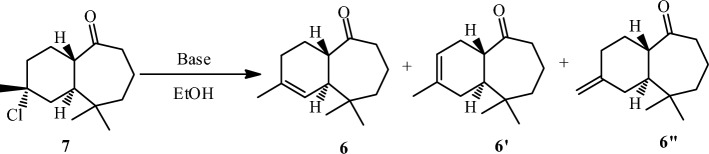
Dehydrohalogenation of himachalone monohydrochloride (7).

With the desired compound (**7)** in hand, the next goal was the synthesis of ketone derivative (**6)** (Scheme 6).

Thus, treatment of (**7**) under typical dehydrochlorination conditions, in the presence of sodium ethoxide in ethanol as solvent, provided a mixture of tree ketones (**6**, **6’** and **6″**). In order to direct the reaction towards the ketone **6**, different parameters that can influence the course of the reaction have been studied such as the nature of the base, the amounts of base used, and the effect of temperature on the reaction. The obtained results are summarized in Table 1.

**Table 1:** Optimization of dehydrohalogenation reaction for product **(6).**

Entry	Bases	base eq	Temperature	Conversion	Selectivity to (6)	Selectivity to (6’ + 6″)
1	MeONa	2	70	100	72	28
2	t-BuOK	2	70	100	73	27
3	AcONa	2	70	100	69	31
4	EtONa	2	70	100	72	28
5	EtONa	1	70	100	71	29
6	EtONa	4	70	100	77	23
7	EtONa	8	70	100	79	21
8	EtONa	12	70	100	82	18
9	EtONa	15	70	100	85	15
10	EtONa	15	25	100	12	2
11	EtONa	15	45	100	95	5
12	EtONa	15	90	100	78	22

Conditions: 10 mL of ethanol; 8 h and 0.2 g of himachalone monohydrochloride.

From entries 1 to 4, (Table 1) it appears that the nature of the base has no influence on the selectivity of the reaction since all the tested bases give almost the same results. To optimize the other reaction conditions, we chose EtONa as a base, then we studied the effect of different amounts of this base and the effect of the reaction temperature. The results show that the selectivity toward the desired product increases with increasing the amount of EtONa at 70 °C (entries 4–9, Table 1). In addition, we noticed that the temperature has an important effect on the selectivity of this reaction (entries 9–12, Table 1). From this study, we have found that the optimal reaction conditions to obtain the ketone as the major product are the use of 15 equivalents of the base at 45° C (entry 11).

The obtained product underwent purification using a silica gel column, employing an eluent composed of a hexane/ethyl acetate mixture in a ratio of 95/5. Subsequently, the purified product was characterized using spectroscopic techniques including ^1^H, ^13^C, HSQC, COSY NMR, MS, and IR.

**Figure 12:**
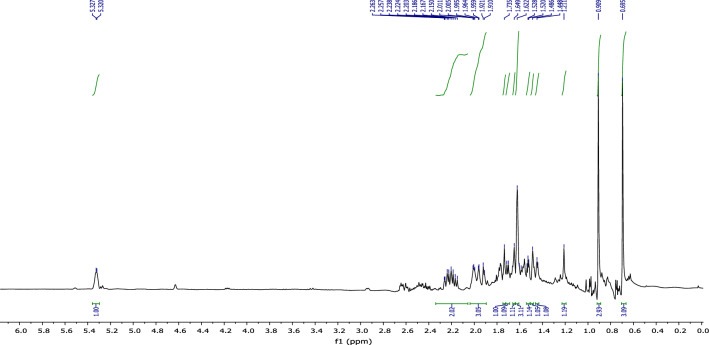
NMR ^1^H (CDCl_3_) of himachalone (6).

In the ^1^H NMR spectrum (Fig. 12), we observe:

• Three signals at 0.70 ppm, 0.91 ppm, and 1.61 ppm, corresponding to the characteristic methyl groups protons.

• A signal in the form of a doublet corresponds to the proton of the double bond at 5.35 ppm with J_3_ = 3 Hz. This multiplicity indicates that the position of the double bond is consistent with the structure of product (**6**).

**Figure 13:**
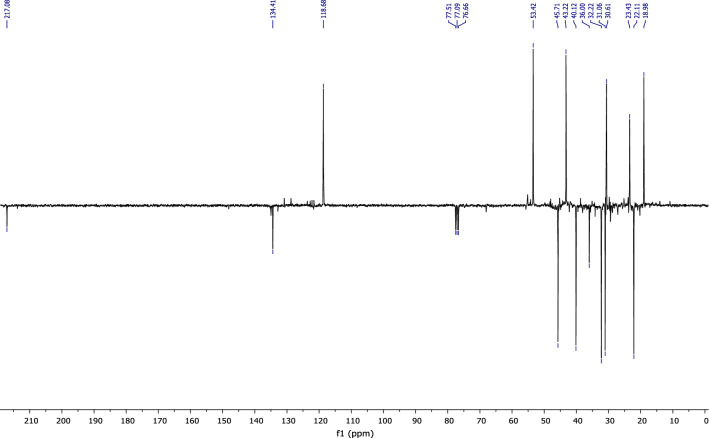
NMR ^13^C (CDCl_3_) of himachalone (6).

According to the ^13^C NMR spectrum using APT (Fig. 13) pulse sequence, we clearly observe the fourteen characteristic peaks of the molecule, notably: the appearance of the peak corresponding to the quaternary carbon and the tertiary carbon of the carbon–carbon double bond at 135 ppm and 110 ppm, respectively.

**Figure 14:**
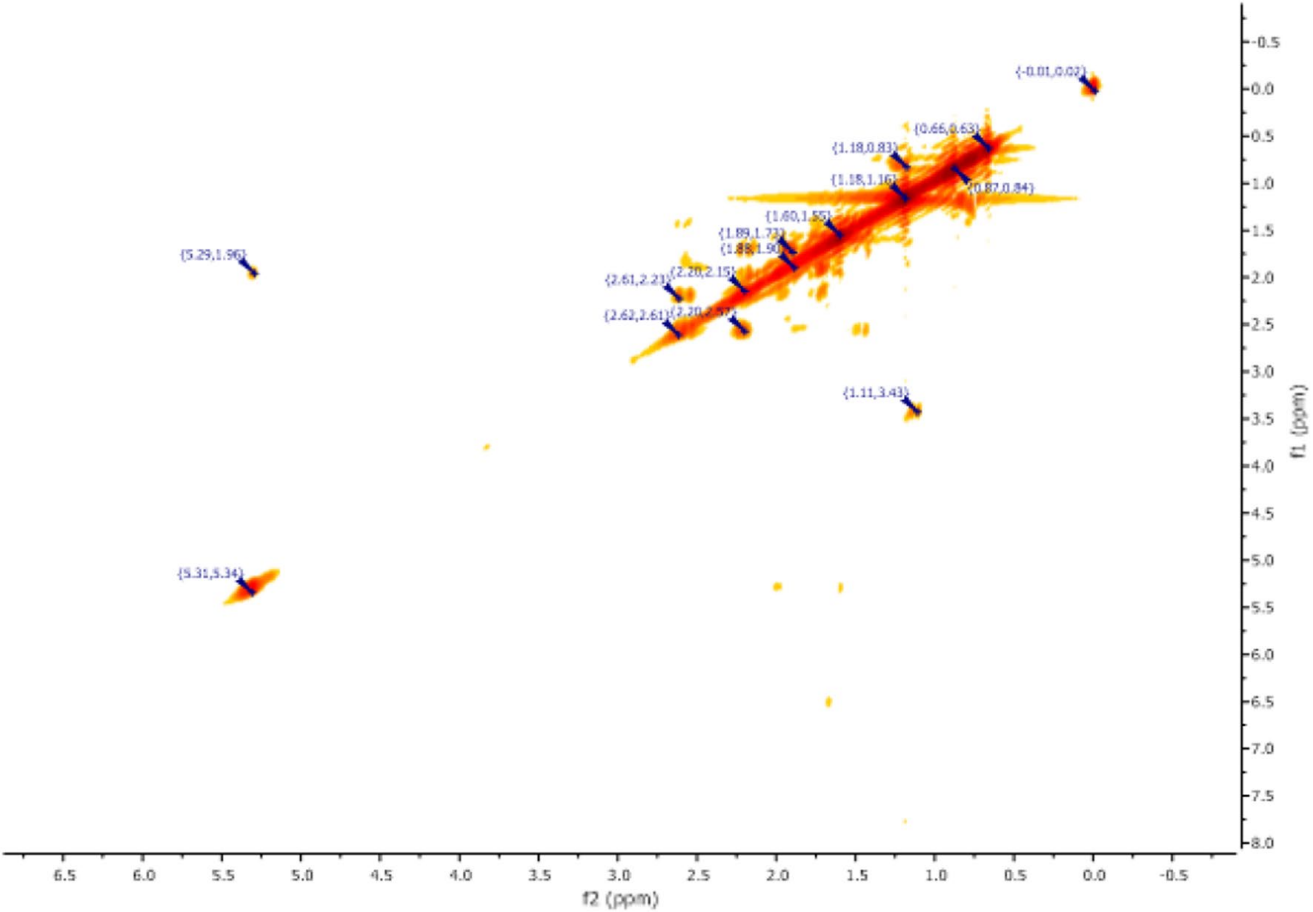
NMR 2D COSY (CDCl_3_) of himachalone (6).

**Figure 15:**
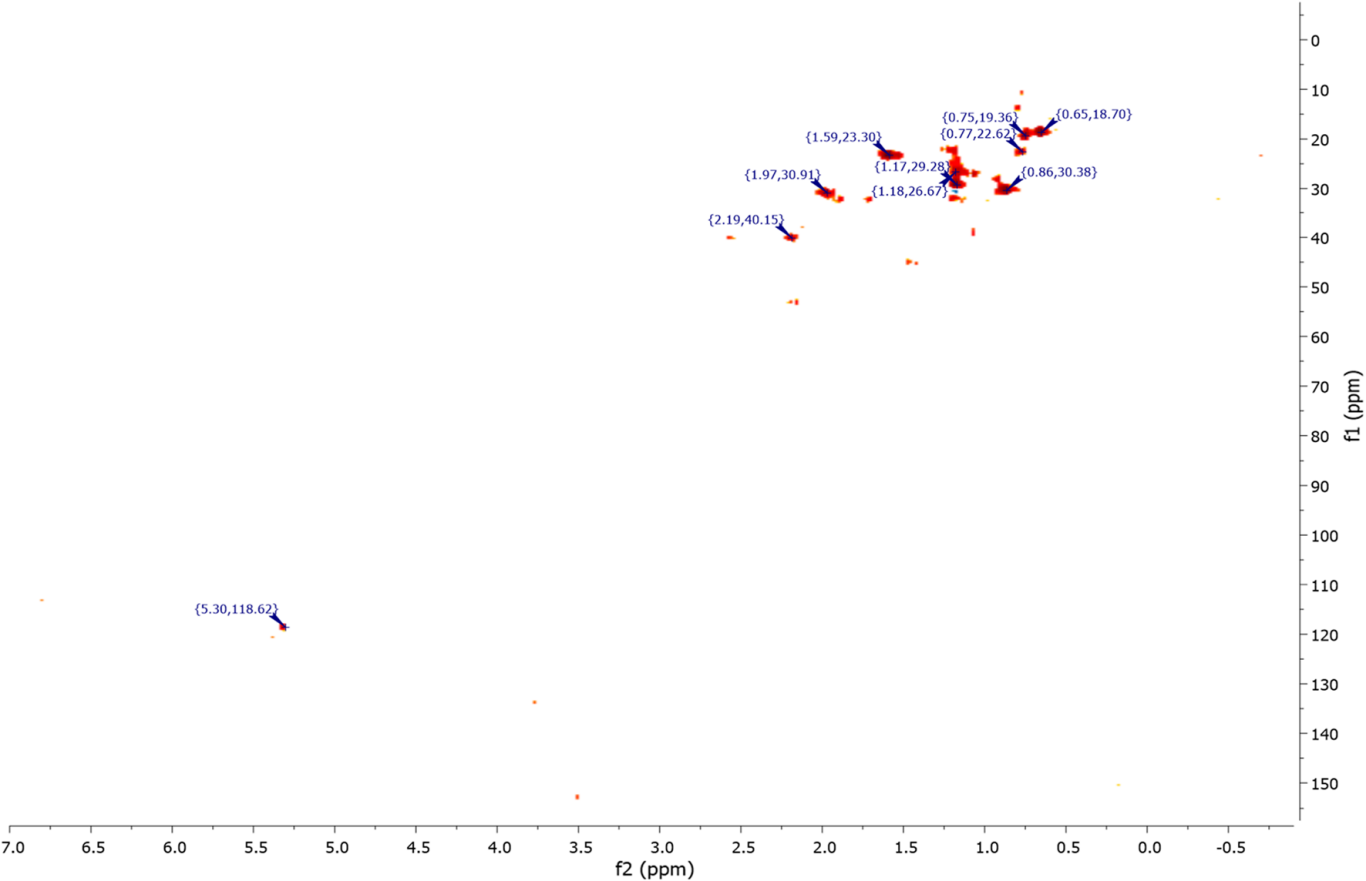
NMR 2D HSQC (CDCl_3_) of himachalone (6).

The results obtained from 2D NMR experiments using COSY pulse sequences (Fig. 14) and HSQC (Fig. 15) have enabled us to correlate the positions of protons with their corresponding carbons. Specifically, these experiments have unveiled crucial information. For instance, the proton located within the double bond was detected at a chemical shift of 5.30 ppm in the HSQC spectrum (Fig. 14), and its associated carbon was identified at 118.62 ppm in the same HSQC (Fig. 15). It is noteworthy that this proton exhibits interaction with another proton at 1.96 ppm, as indicated in the COSY spectrum (Fig. 14). This latter proton happens to be the closest to the former and is positioned at the intersection of the two cycles.

**Figure 16:**
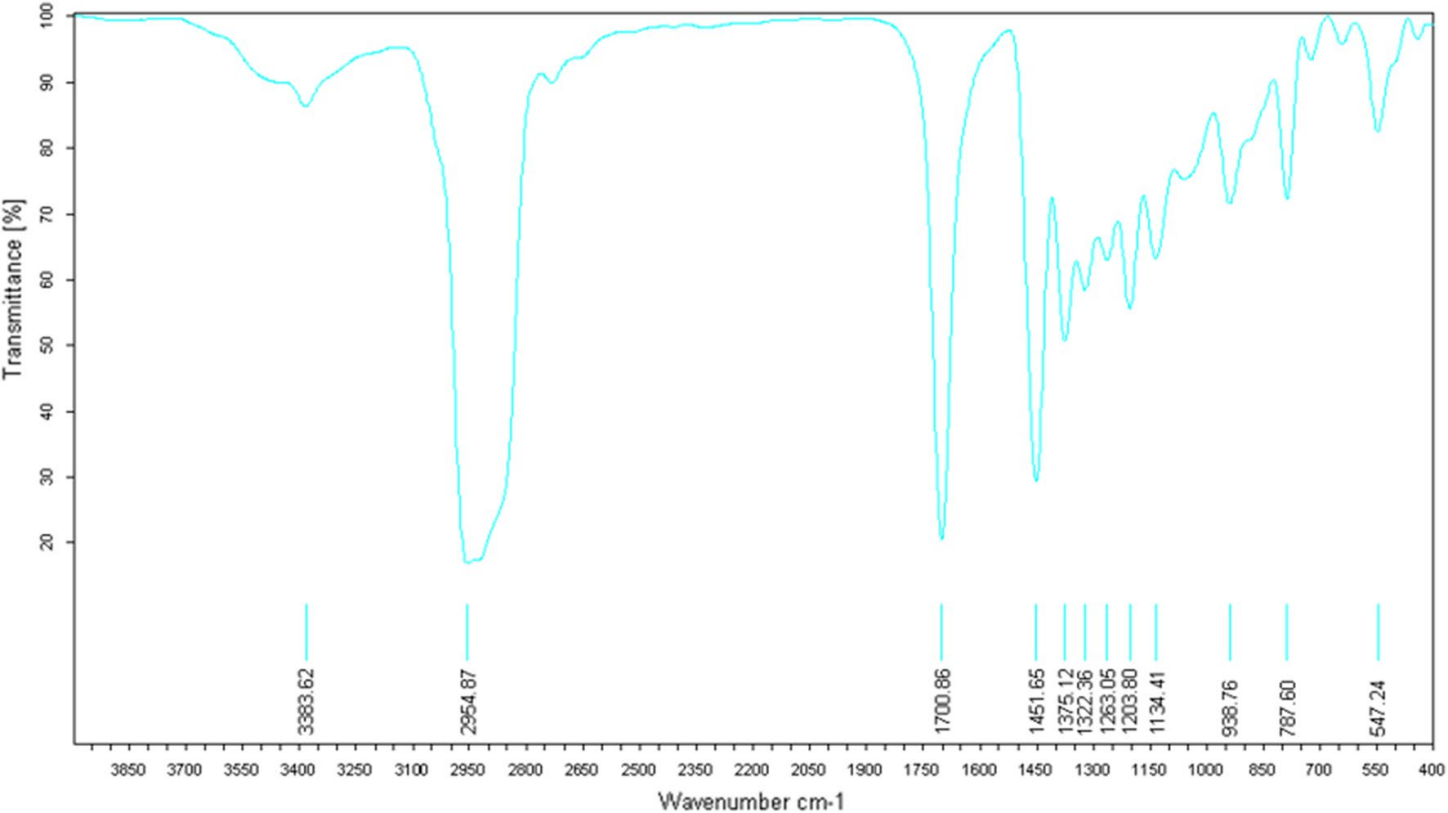
IR spectrum of himachalone (6).

The IR analysis (Fig. 16) reveals a notably broad and intense band characteristic of the ketone functional group at 1700.86 cm^−1^.

**Figure 17:**
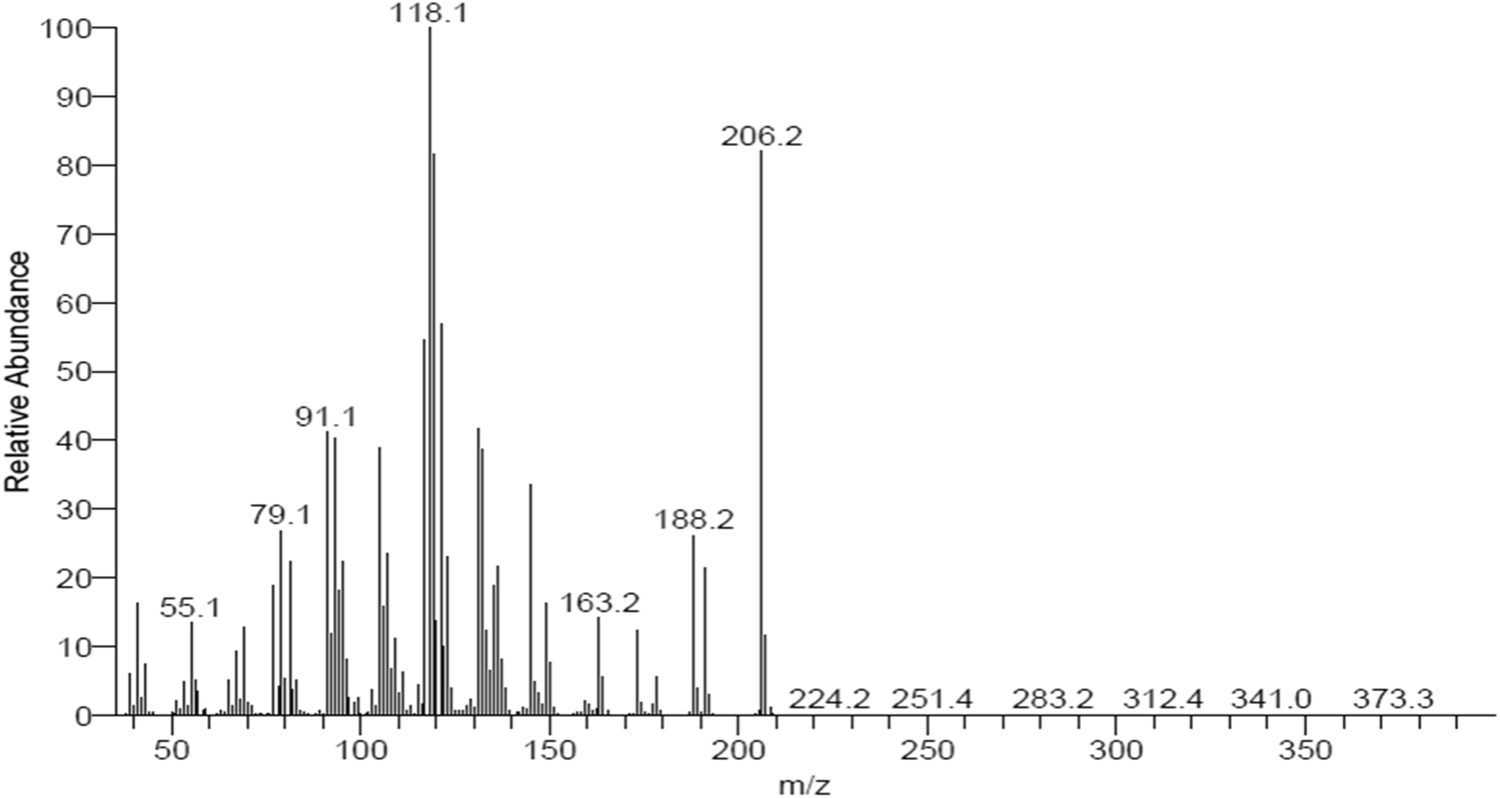
mass spectrum of himachalone (6).

Gas Chromatography-Mass Spectrometry (GC–MS) (Fig. 17) analysis below reveals that the molecular mass of himachalone is m/z = 206.2.

**Scheme 7:**
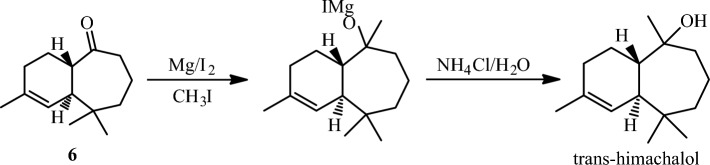
Preparation of trans-himachalol according to Grignard method.

In the last step of the synthesis of trans-himachalol, we applied the Grignard method to transform the carbonyl function of ketone (**6)** into tertiary alcohol by the use of CH_3_MgI as a Grignard reagent according to the scheme described below (Scheme 7). The reaction lead to trans-himachalol (**5**) in a 96% yield.

The obtained product is purified using a silica gel column chromatography eluted with hexane/ethyl acetate (95/5) and then characterized by ^1^H, ^13^C NMR, 2D NMR (COSY, HSQC), mass and IR spectroscopy.

**Figure 18:**
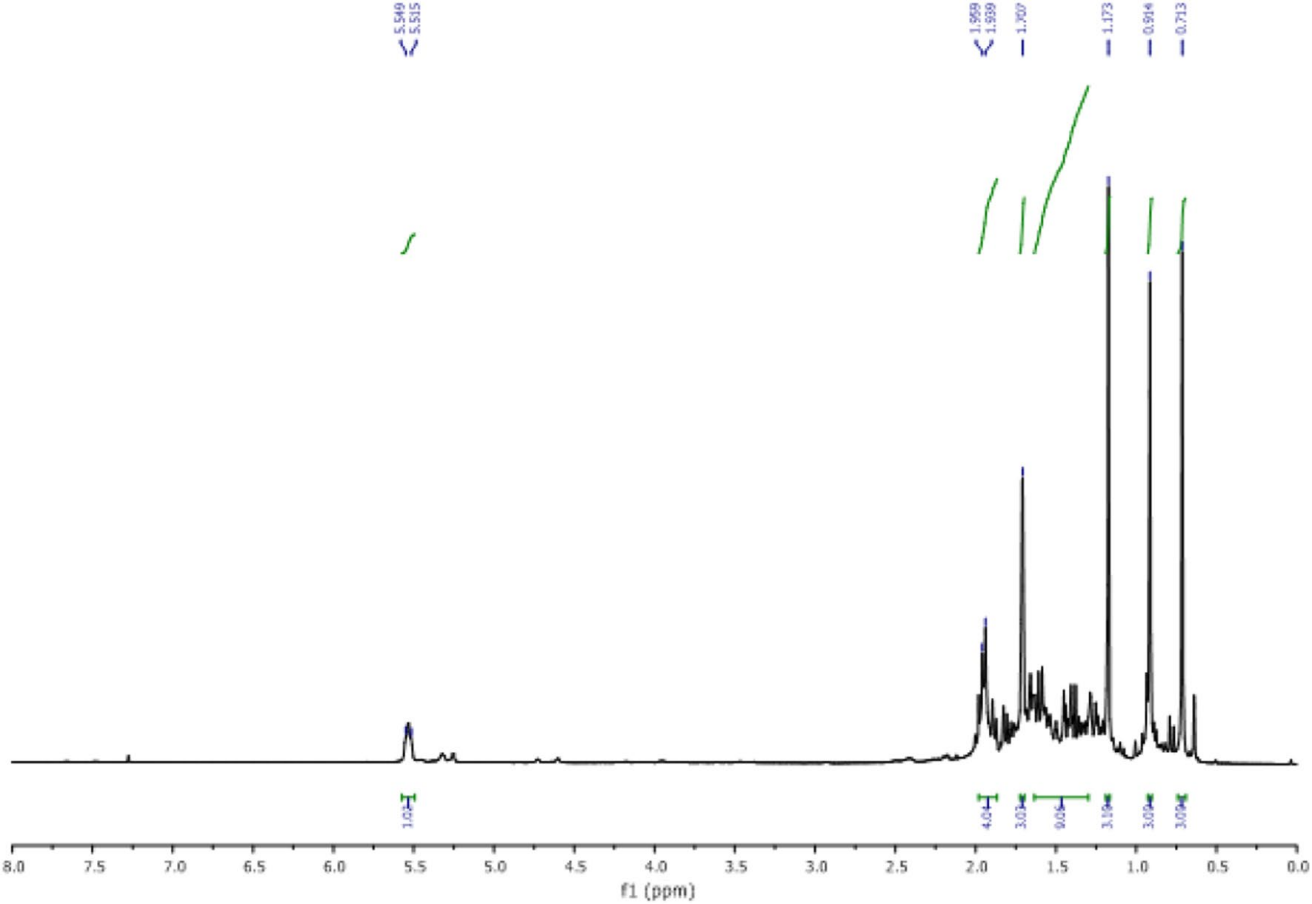
NMR ^1^H (CDCl_3_) of himachalol (5).

In the ^1^H NMR spectrum (Fig. 18)., we specifically observe:

• Four signals at 0.71 ppm, 0.91 ppm, 1.17 ppm, and 2.75 ppm, corresponding to the characteristic methyl groups protons.

• A signal in the form of a doublet corresponds to the proton of the double bond at 5.51 ppm.

**Figure 19:**
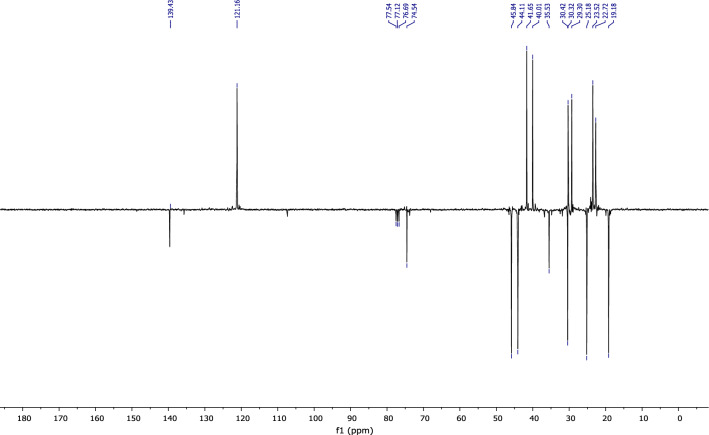
NMR ^13^C (CDCl_3_) of himachalol (5).

According to the ^13^C NMR spectrum using APT pulse sequence, we clearly observe the fourteen characteristic peaks of the molecule, notably: the appearance of the peak corresponding to the carbon of the new methyl group at 30 ppm and the disappearance of the peak of the carbonyl carbon (Fig. 19).

**Figure 20:**
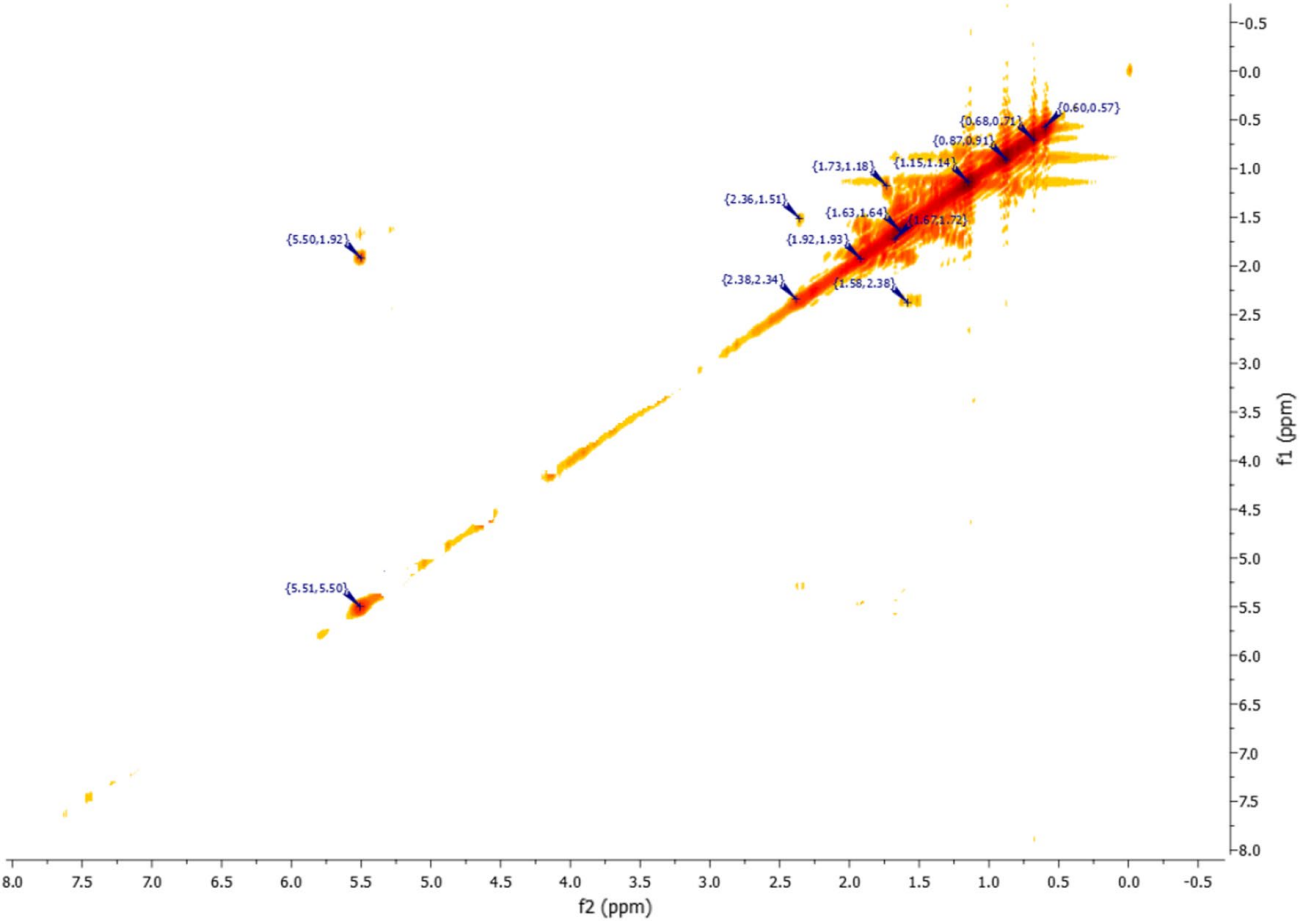
NMR 2D COSY (CDCl_3_) of himachalol (5).

The conclusions drawn from the 2D NMR experiments using COSY pulse sequences (Fig. 20) specifically highlight that the proton located in the double bond is detected at a chemical shift of 5.35 ppm and exhibits interaction with a proton at 1.96 ppm. This latter proton is the closest to the former and is positioned at the intersection of the two cycles.

**Figure 21:**
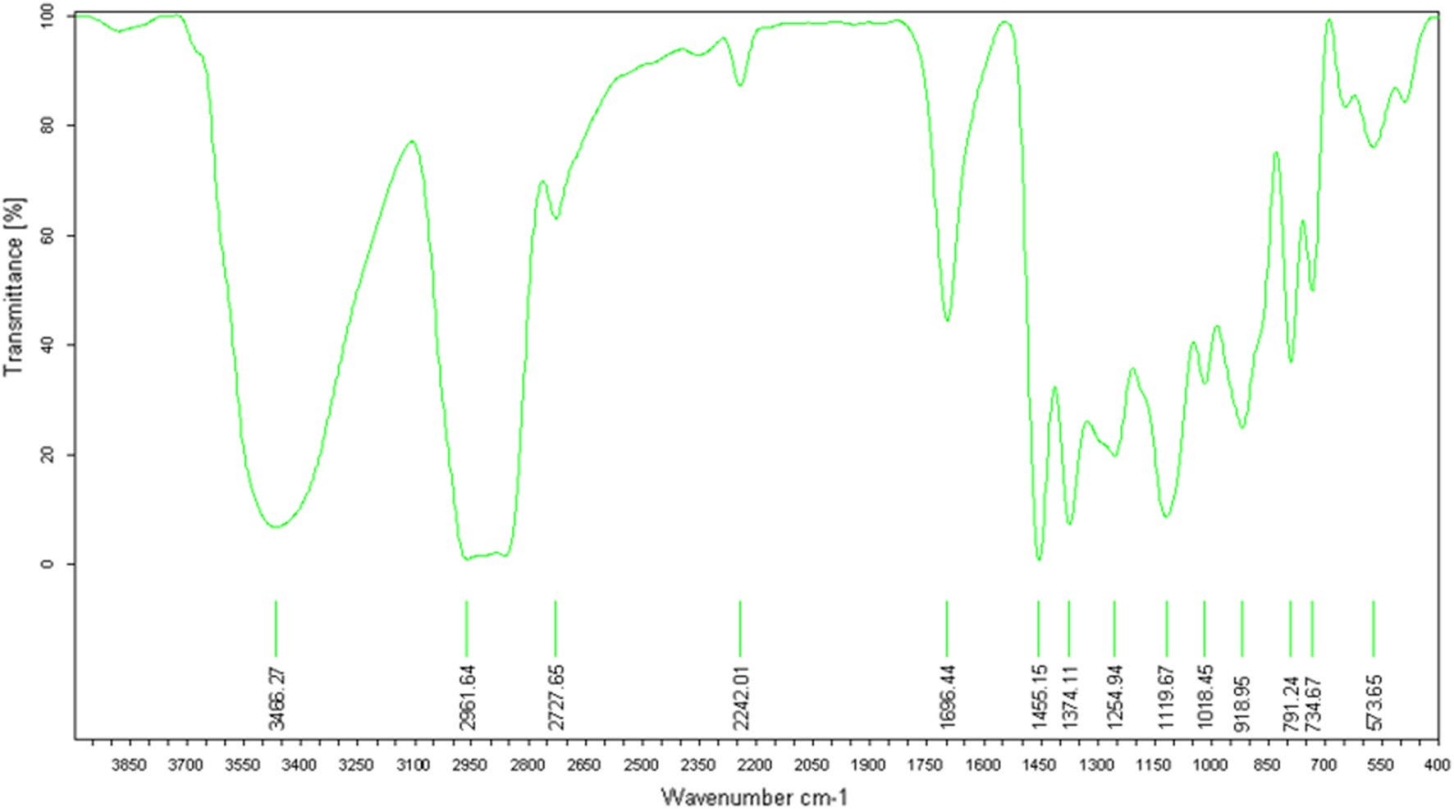
IR spectrum of himachalol (5).

The IR analysis (Fig. 21) specifically highlights two characteristic bands of the alcohol functional group. The first broad and intense band at 3486,27 cm^−1^ corresponds to the O–H bond, while the second narrow band at 1696,44 cm^−1^ corresponds to the C-O bond.

**Figure 22:**
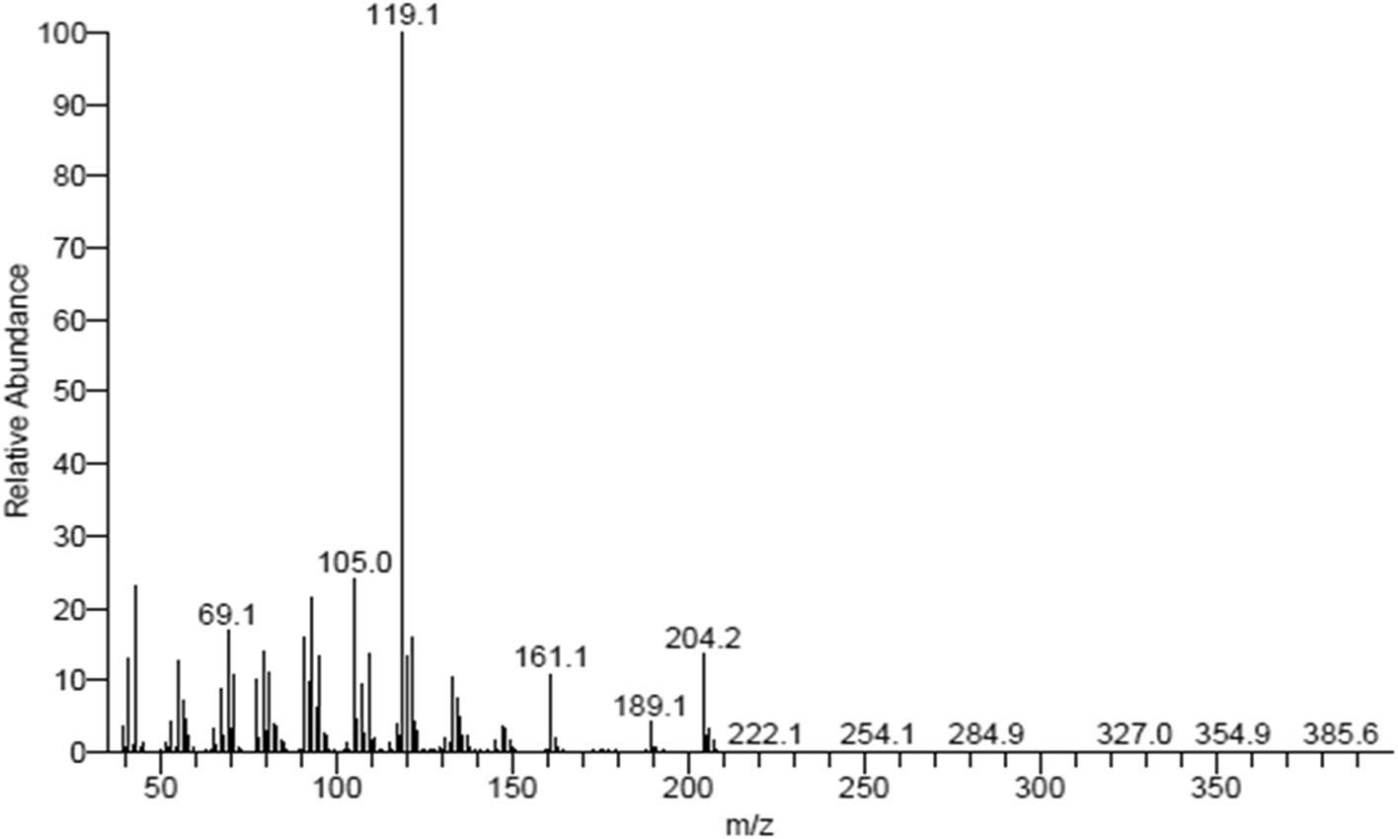
Mass spectrum of himachalol (5).

Gas Chromatography-Mass Spectrometry (GC–MS) analysis below reveals that the molecular mass of himachalol is m/z = 222.1 (Fig. 22).

### In silico ADME predictions

Due to its cost-effectiveness and capacity for high throughput, ADME modeling has gained popularity in pharmaceutical research for drug development. Tables 2 and 3 present the outcomes of Lipinski's rule of five and the ADME predictions from Qikprop for the primary compounds derived from himachalenes and the positive control. The analysis revealed that all the studied compounds, including molecular weight (under 500 g/mol), hydrogen bond donors (not exceeding 5), hydrogen bond acceptors (not exceeding 10), and the anticipated octanol/water partition coefficient (less than 5), were accurately predicted to adhere to Lipinski's rule of five without any violation.

**Table 2:** In silico Lipinski's rule of five for main compounds derived from himachalenes.

Compound name	Molecular weiǥht	Donor HB	Acceptor HB	QPlogPo/w	Rule of five
Trans-himachalol **(5)**	222.37	1	0.75	3.929	0
Himachalone **(6)**	206.32	0	2	3.172	0
Himachalone monohydrochloride **(7)**	242.78	0	2	3.670	0
Physostigmine	275.35	1	6	1.263	0

**Table 3:** Predicted ADME analysis for main compounds derived from himachalenes.

Compound name	SASA	FOSA	FISA	PISA	Volume	QPPCaco	QPlogBB	HOA
Trans-himachalol **(5)**	466.5	420.4	32.2	13.9	833.0	4901.8	0.248	3
Himachalone **(6)**	444.9	393.4	41.1	12.3	784.9	4036.3	0.240	3
Himachalone monohydrochloride **(7)**	457.8	362.7	38.8	0.00	828.3	4240.2	0.403	3
Physostigmine	535.0	377.3	69.1	88.5	938.2	938.2	0.686	2

SASA: Solvent Accessible Surface Area (Range 300.0–1000.0).

FOSA: Hydrophobic Component of SASA (Range 0.0–750.0).

FISA: Hydrophilic Component of SASA (Range 7.0–330.0).

PISA: Pi Component of SASA (Range 0.0–450.0).

Volume: Total solvent-accessible volume in cubic angstroms (Range 500.0–2000.0).

QPPCaco: Predicted apparent Caco-2 cell permeability in nm/sec (Range < 25 poor, > 500 great).

QPlogBB: Predicted brain/blood partition coefficient (Range − 3.0–1.2).

HOA: Predicted qualitative human oral absorption (Range 1, 2, or 3 for low, medium, or high).

All compounds derived from himachalenes, including trans-himachalol (5), himachalone (6), and himachalone monohydrochloride (7), were thoroughly examined. Their values were found to fall within the range stipulated in the Schrödinger's Qikprop manual for investigating surface components, which include SASA, FOSA, FISA, PISA and volume. Moreover, the qualitative human oral absorption was predicted to be high for all compounds, with QPPCaco (donner le nom complet avant d’utiliser l’abbréviation) values exceeding 500 nm/sec, indicating excellent permeability. Additionally, the QPlogBB value fell within an acceptable range between -3.0 and + 1.2. Regarding the Volume, all compounds, exhibited values within the favorable range of 500–2000. In summary, the main compounds derived from himachalenes displayed favorable values in accordance with Lipinski's rule of five and predicted pharmacokinetic parameters, with no violations, demonstrating drug-like characteristics. This comprehensive analysis holds significant importance in advancing these compounds as potential pharmaceutical agents and in exploring their potential applications in the treatment of relevant medical conditions.

### Molecular docking results

**Figure 23:**
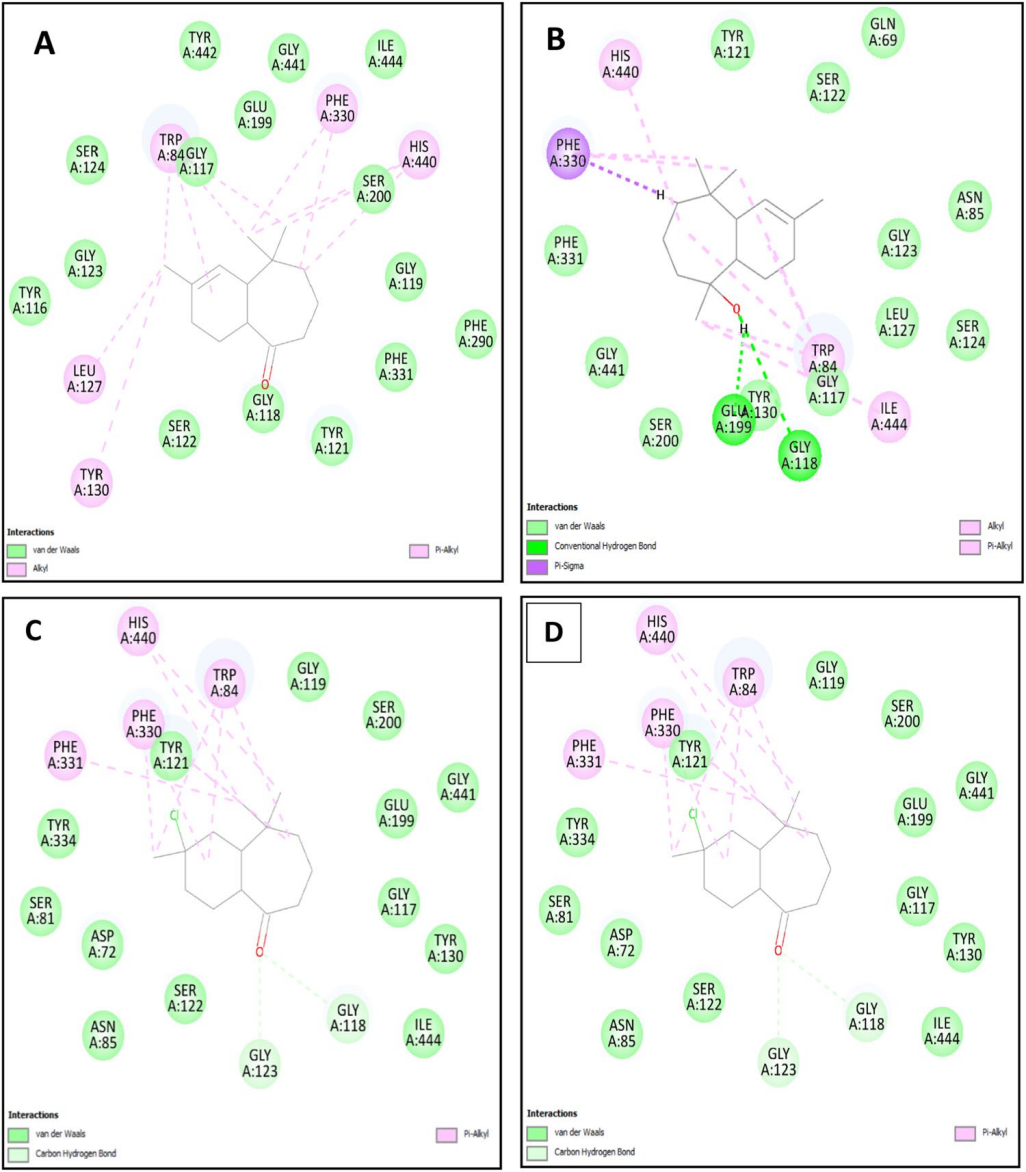
Intermolecular interactions 2D between (**A**) trans-himachalol (**5**), (**B**) himachalone (**6**), (**C**) himachalone monohydrochloride (**7**) and (**D**) control Physostigmine with the 7B2W protein.

Molecular docking is a significant technique in silico drug design and detection procedures, allowing the assessment of the interaction between a molecule and a receptor through binding affinity score^27–29^. In this study, we investigated potential compounds synthesized from himachalenes (trans-himachalol (**5**), himachalone (**6**), and himachalone monohydrochloride (**7**)) using molecular docking, and they exhibited promising activities on various isolated smooth muscles and against different neurotransmitters. The strength of the ligand-receptor interaction was evaluated using docking scores, where a more negative score indicates a higher binding affinity between the targets and ligands (Table 4)^21^. The results indicated that the compounds trans-himachalol (**5**), himachalone (**6**), and himachalone monohydrochloride (**7**) displayed high affinities towards the active site of the protein 7B2W, with estimated binding energy values of -7.137 kcal/mol, − 7.049 kcal/mol, and -6.819 kcal/mol, respectively. These values were notably better than the binding energy of Physostigmine (positive control) used in this study, which was just − 3.959 kcal/mol. Further analysis was conducted to investigate the interaction modes of the more stable selected compound, trans-himachalol (**5**), within the active site of the protein and to elucidate the inhibition mechanism. As shown in Fig. 23, trans-himachalol (**5**), which exhibited the lowest binding energy and is considered the most probable active inhibitor, interacted with surrounding residues through various interactions. It formed conventional hydrogen bonds with two amino acids, GLU199 and GLY130, as well as a Pi-sigma interaction with PHE330. Additionally, it was stabilized by two Pi-alkyl interactions with HIS440 and TRP84, and three alkyl bonds with PHE330, TRP84, and ILE444. Moreover, the residues TYR121, SER122, GLN69, ASN85, GLY123, SER124, LEU127, GLY117, TYR130, SER200, GLY441, and PHE331 were implicated in van der Waals interactions. Further experimental validations are required to assess and gain a comprehensive understanding of the therapeutic effects of these compounds, which have demonstrated promising activities on various isolated smooth muscles and have exhibited effectiveness against different neurotransmitters. The results obtained from molecular docking studies provide valuable insights, but to establish their practical applications as potential therapeutic agents, in vitro and in vivo experiments are essential. These validations will enable researchers to evaluate the compounds' interactions with their target receptors or enzymes, study their pharmacokinetic properties, and assess their safety and efficacy profiles. This thorough investigation is crucial for the development of these compounds as potential drugs and to advance their potential application in treating relevant medical conditions.

**Table 4:** The docking scores and the binding residues of the selected compounds against the 7B2W protein.

Compound name	Docking scores (kcal/mol)	Contribution to major binding residues
Trans-himachalol **(5)**	− 7.137	GLU199-GLY130-PHE330-TRP84-ILE444-HIS440
Himachalone **(6)**	− 7.049	TRY130-LEU127-TRP84-PHE330-HIS440-TRP84
Himachalone monohydrochloride **(7)**	− 5.798	GLY118-GLY123-PHE331-PHE330-HIS440-TRP84
Physostigmine	− 3.959	TRP84-TYR121-GLY118-HIS440-TYR334-PHE330-TRP84-HIS440

## Conclusion

In conclusion, a total synthesis of trans-himachalol (**5**), a bicyclic sesquiterpene, has been achieved in five steps starting from himachalenes. This project is part of the valorization of natural compounds derived from Moroccan resources. The regioselective hydrochlorination reaction represents the key step in obtaining the target product (**6**). The structure of trans-himachalol was successfully characterized using various spectroscopic techniques. Molecular docking study shows that the synthesized compounds exhibit promising activities on various isolated smooth muscles and against different neurotransmitters.

## Data Availability

Adequate and clear descriptions of the applied materials and tools are provided in the materials and method section of the manuscript. In addition, the obtained data is justifed by mentioning the figures and tables in the manuscript.
